# Availability and affordability of medicines for children

**DOI:** 10.1186/2052-3211-8-S1-P10

**Published:** 2015-10-05

**Authors:** Irina Kazaryan, Lusine Vardanyan

**Affiliations:** 1 Department of Pharmaceutical Management, Yerevan State Medical University, Yerevan, 0025, Armenia

## Background

Better access to essential medicines for children is an important pre-condition for improving their health and reducing mortality. However, the results of surveys implemented in some countries show that treatment of children is not always affordable due to a high cost of medicines. The objective of this work was to assess the situation with availability, prices and affordability of essential medicines for children in Armenia.

## Methods

Data collection and analysis was conducted using an adaption of the standardized methodology developed by the World Health Organization and Health Action International (Better Medicines for Children project). Data on availability of 37 paediatric medicines were collected from 33 private medicine outlets from all regions of Armenia (in 2013). Full patient prices of available medicines were recorded. Affordability was expressed as the number of days needed by a person who earns the minimum wage that was set by the legislation, to purchase a course of treatment described in clinical guidelines approved by the Ministry of Health.

## Results

Only 13 of 37 (35.1%) paediatric medicines were available at medicine outlets. Originator brands were available for three of 13 medicines, generics, for all 13 pharmaceuticals. The mean availability of originator brands (OBs), highest priced generics (HPGs) and lowest priced generics (LPGs) were 20.5%, 52.9 % and 43.0%, correspondingly. Only three of 13 LPGs were found at more than 80% of medicine outlets. Considerable difference was observed between prices of HPG and LPGs - on average prices of OBs were 7.4 times the price of LPGs. Affordability was measured for those six of 13 available medicines which were identified in paediatric clinical guidelines. As original brands for these six medicines were not available, affordability was calculating for lowest and highest priced generics. Cost of a course of pneumonia treatment ranged from 1.7 days' wages for amoxicillin suspension (lowest priced generic) to 4.8 days' wages for ceftriaxone injection (highest priced generic). For treating bacterial infection those on the minimum wage would have to pay for benzylpenicillin injection from 1.9 (lowest priced generic) to 6.3 days' wages (highest priced generic). Purchasing a salbutamol inhaler to treat asthma required 0.7 days' wages when using the lowest priced generic and 1.9 days' wages when using the highest priced generic.

## Conclusions

The availability of paediatric medicines was low. Treatment can be unaffordable for those children who are not covered by the reimbursement system. Urgent interventions should be identified and implemented, including price control measures.

